# Causes of Death and Conditional Survival of Renal Cell Carcinoma

**DOI:** 10.3389/fonc.2019.00591

**Published:** 2019-07-15

**Authors:** Ning Shao, Fangning Wan, Mierxiati Abudurexiti, Jun Wang, Yao Zhu, Dingwei Ye

**Affiliations:** ^1^Department of Urology, Fudan University Shanghai Cancer Center, Shanghai, China; ^2^Department of Oncology, Shanghai Medical College, Fudan University, Shanghai, China

**Keywords:** conditional survival, renal cell carcinoma, causes of death, follow-up, high-risk

## Abstract

**Background:** As conditional survival could provide more relevant prognostic information at each follow-up time, the present study aimed to assess conditional overall survival (COS) based on two cohorts and assess the risks of death due to renal cell carcinoma (RCC) vs. other causes.

**Methods:** The Fudan University Shanghai Cancer Center (FUSCC) and Surveillance, Epidemiology, and End Results (SEER) database were used as the source of data for our analysis. COS and cancer-specific survival were evaluated using the Kaplan–Meier method.

**Results:** A total of 90,927 patients (SEER cohort = 88,807, FUSCC cohort = 2,120) were enrolled. Our results suggest that hazards of other causes-related death were always higher than that of cancer-specific death in low-risk RCC patients, but lower in metastatic RCC patients. It exceeded that of cancer-specific death by 8 years in high-risk RCC patients. Only in metastatic RCC patients, the COS improved markedly with survivorship increasing. After surviving 1, 2, 3, 4, and 5 years, the 5 years COS increased by +10, +18, +23, +29, and 35% (the observed 5 years OS: 12%), respectively.

**Conclusions:** COS can better help patients with metastatic RCC rather than other RCC patients. Additionally, COS brings optimism for metastatic RCC patients with expected poorer prognosis psychologically.

## Introduction

Renal cell carcinoma (RCC) is the most common kidney cancer. Nearly, 70−80% patients with RCC have clear cell histology, and non-clear cell RCC (non-ccRCC) encompasses the remaining tumors (1, 2). Seventy percent of RCC patients have no metastatic RCC (mRCC) at the time of initial diagnosis, while 20% of patients will relapse and develop mRCC after nephrectomy (3, 4). The National Comprehensive Cancer Network (NCCN) guidelines suggested that the median time to relapse after surgery was 1–2 years, with most relapse occurring within 3 years (5). Additionally, NCCN recommends that the longest surveillance duration for most RCC patients is 5 years.

However, 5 year survival does not equate with a definitive cure. Traditional overall survival (OS) could not reflect individual prognosis accurately after initial disease management. Conditional survival (CS), derived from the concept of conditional probability, could provide more relevant prognostic information at each follow-up time (6–8). Although some previous studies reported the CS of RCC with small sample size, it was difficult to identify higher-risk patients who require longer follow-up. The NCCN suggested that further research was required to refine follow-up strategies for these patients.

Hence, the aim of the present study was to assess conditional OS (COS) based on two cohorts with longer-term follow-up and quantify yearly risks of death due to RCC or other causes. We then investigated the COS and cancer-specific survival (CSS) in different risk groups and histologic subtypes using a competing risk model for the development of specific follow-up strategies. These analyses have important implications for patient counseling and follow-up planning.

## Materials and Methods

### Patients

#### SEER Cohort

The Surveillance, Epidemiology, and End Results (SEER) database was used as the main source of data for our analysis. Data from SEER database were retrieved from 2004 to 2014. Only patients with microscopically confirmed RCC (using ICD-O-3 histology/behavior codes: 8260/3, 8270/3, 8290/3, 8310/3, 8312/3, 8316/3, 8317/3, 8319/3, 8320/3, 8323/3, 8480/3, and 8510/3) were included. The other variables like year of diagnosis, cause of death, age at diagnosis, race/ethnicity, staging information, follow-up data, and sex were also collected. Deaths were classified as due to RCC or other causes.

#### FUSCC Cohort

The data of patients with RCC from Fudan University Shanghai Cancer Center (FUSCC cohort) were obtained from FUSCC (from 2000 to 2018). Our study was approved by the Ethics Committee of FUSCC. The written informed consent were obtained from the patients. All included patients have been histologically confirmed by surgery or biopsy in our department. Patients with RCC in our cohort were staged according to the definitions of the 8th American Joint Committee on Cancer (AJCC) stage grouping by using abdominal/pelvic computed tomography (CT) scan or magnetic resonance imaging (MRI) when needed. After informed consent was obtained, patients were well-informed of the importance of follow-up. Patients were regularly followed up every 3 months for the first 3 years, then every 6 months up to 5 years, then annually thereafter.

### Statistical Analyses

Data analysis was conducted from October 10 to November 15, 2018. Patients were censored if they were lost to follow-up or died. COS and CSS were evaluated using the Kaplan–Meier method. Subgroup analyses were also performed according to diverse pathological RCC or different risks. According to the NCCN guidelines, risks were classified as low risk (stage I and II), high risk (stage III or higher, regional lymph node metastatic, or both), and mRCC. Smoothed yearly hazards of death due to RCC, other causes, and any cause were evaluated graphically. CS is the proportion surviving. For example, five additional years, per the following equation: when *S*(*t*) is OS at time *t*, CS is *S*(*x* +5)/*S*(*x*). Standardized differences (*d*) were used to assess the differences of CS between subgroups based on the method described by Cucchetti et al. (9) and Kim et al. (10). The standardized difference in proportions is calculated as (P2–P1)/√[*P*(1–*P*)], where *P* is the weighted mean of P1 and P2:

1. *d* values lower than |0.1| indicate very small differences between means;
2. *d* values between |0.1| and |0.3| indicate small differences,
3. *d* values between |0.3| and |0.5| indicate moderate differences,
4. and *d* values > |0.5| indicate considerable differences.

All statistical analyses were performed using R (version 3.4.2, www.r-project.org). All statistical tests were two sided, and a *P* < 0.05 was considered statistically significant.

## Results

### Cohort Characteristics

A total of 90,927 patients (SEER cohort = 88,807, FUSCC cohort = 2,120) were enrolled for the CS analysis. Of these patients, 33,428 (36.76%) patients were female and 72,812 (80.08%) were from majority (white) populations. The median age at diagnosis was 60.84 years. Table 1 summarizes the demographic and clinical characteristics of the patients in both cohorts. The proportions of male patients, young patients (<65), low-risk patients, and ccRCC patients in the FUSCC cohort were higher.

**Table 1:** Demographic and clinical characteristics of the SEER and FUSCC cohorts.

**Characteristics**	**SEER cohort**	**FUSCC cohort**
	***n* = 88,807**	***n* = 2,120**
**AGE, YEARS**		
<65	53,096 (59.79%)	1,633 (77.03%)
≥65	35,711 (40.21%)	487 (22.97%)
**SEX**		
Male	50,649 (57.03%)	1,450 (68.40%)
Female	32,758 (42.97%)	670 (31.60%)
**8TH AJCC PROGNOSTIC STAGE**		
I	55,177 (62.13%)	1,493 (70.42%)
II	8,654 (9.74%)	190 (8.96%)
III	12,722 (14.32%)	233 (10.99%)
IV	12,254 (13.81%)	204 (9.62%)
**HISTOPATHOLOGIC TYPE**		
ccRCC	50,739 (57.13%)	1,740 (82.08%)
Papillary RCC	9,834 (11.07%)	89 (4.20%)
Chromophobe RCC	4,776 (5.38%)	82 (3.87%)
Collecting duct RCC	227 (0.26%)	6 (0.28%)
Renal medullary carcinoma	47 (0.05%)	15 (0.71%)
Other RCC	17,494 (26.11%)	188 (8.87%)

### Cause of Death

**Figure 1:**
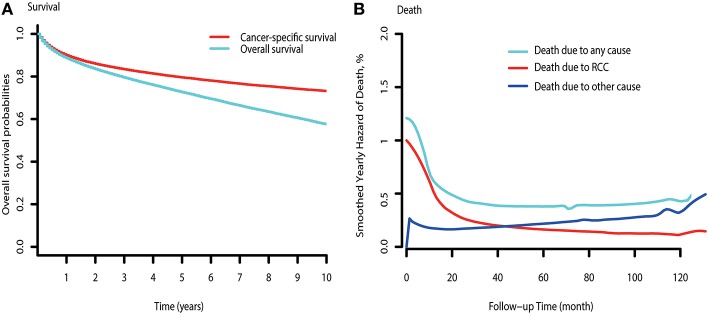
Survival curve and hazards of death curve of RCC. **(A)** CSS and OS curve of RCC. **(B)** Yearly hazards of death due to RCC, other causes, and any cause.

The observed overall CSS and OS are shown in Figure 1A. Hazards of RCC-related death peaked at the beginning and diminished onward (Figure 1B). Hazards of death due to other causes exceeded that of RCC-related death by ~3.5 years after diagnosis. Subgroup analysis indicated that hazards of death due to other causes were consistently higher than that of RCC-related deaths in low-risk patients (Figure S1A). However, the hazards of other causes-related death were always lower than that of death due to RCC in mRCC patients (Figure S1C). For the high-risk patients, the hazards of death due to other causes approximately exceeded that of RCC-related death by 7.8 years after diagnosis (Figure S1B).

In high-risk patients with different pathological types of RCC (ccRCC, chromophobe RCC, and papillary RCC), similar trends of hazards of death were observed (Figure S1D). Additionally, the time of hazards of death due to other causes exceeding due to RCC in ccRCC patients was nearly 2 years longer than that of chromophobe RCC and papillary RCC patients, which indicated that high-risk ccRCC patients need longer surveillance duration.

Compared with RCC-related death in these different pathological RCC patients, the hazards of death due to other causes were higher in low-risk patients and lower in mRCC patients.

### CS in the SEER Cohort

**Figure 2:**
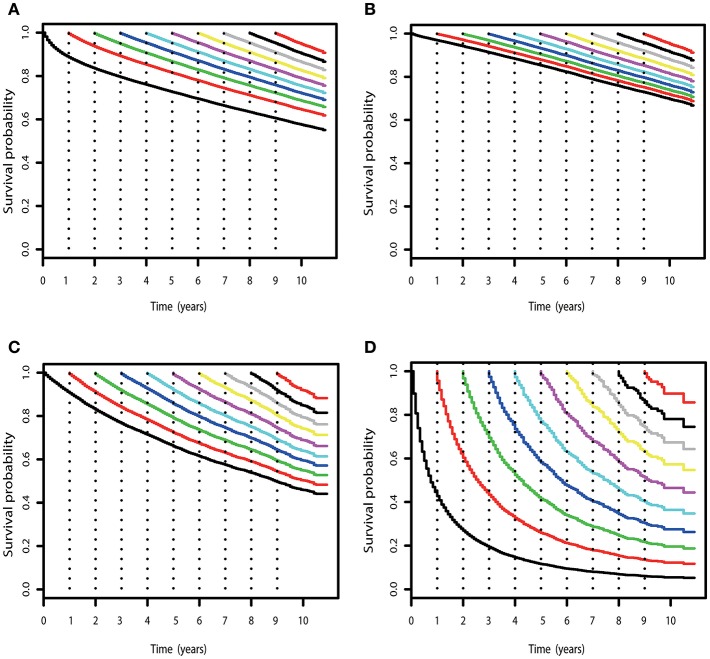
COS curves of RCC. **(A)** COS curves according to the number of years after diagnosis for all patients with RCC in SEER cohort. **(B)** COS curves for patients with low-risk RCC in the SEER cohort. **(C)** COS curves for patients with high-risk RCC in the SEER cohort. **(D)** COS curves for patients with mRCC in the SEER cohort.

**Figure 3:**
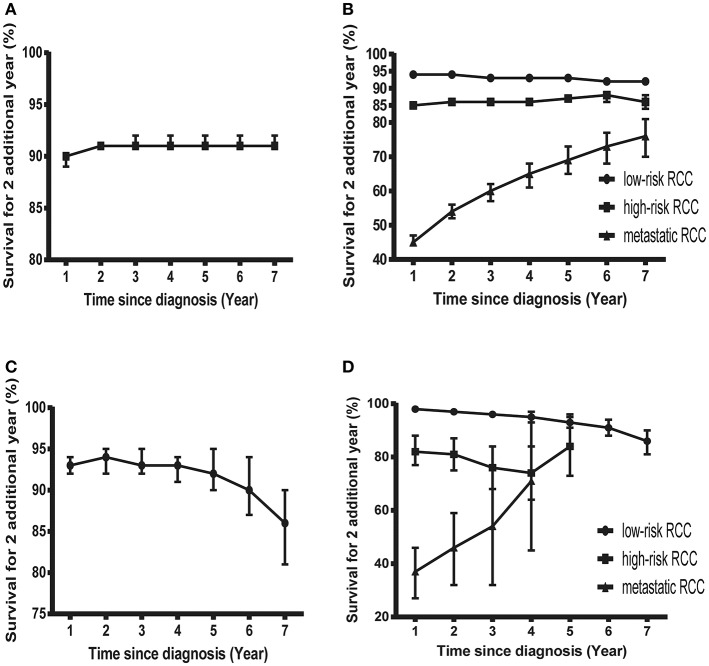
Probability of surviving an additional 2 years in patients with RCC. **(A)** Probability of surviving an additional 2 years at various time points in patients with RCC in the SEER cohort. **(B)** Probability of surviving an additional 2 years at various time points in patients with low-risk, high-risk, and mRCC in the SEER cohort. **(C)** Probability of surviving an additional 2 years at various time points in patients with RCC in the FUSCC cohort. **(D)** Probability of surviving an additional 2 years at various time points in patients with low-risk, high-risk, and mRCC in the FUSCC cohort.

Table 2 gives the COS at various time points for RCC patients treated. Overall, COS keeps a relatively stable level with survivorship increasing (Figures 2A, 3A). For instance, the 2 year COS was almost 91% after living 1, 2, 3, and 4 (or more) years. This indicated that the estimated additional 2 year OS rate for a patient who had lived for 5 years was similar with that of living for 1, 2, 3, or 4 years, but a litter higher than a patient who was recently diagnosed (91 vs. 83%, small differences). In addition, the COS of surviving to 5 years after living 1, 2, 3, and 4 years were 82, 87, 91, and 96%, respectively (higher than the observed 5 year OS: 73%).

**Table 2:** COS at various time points in the SEER cohort.

**Time point**			**COS since time point (months)**											
	**Observed survival**		**12**		**24**		**36**		**48**		**60**		**72**	
	**%**	**95% CI**	**%**	**95% CI**	**%**	**95% CI**	**%**	**95% CI**	**%**	**95% CI**	**%**	**95% CI**	**%**	**95% CI**
12	89	88–89	94	94–94	90	89–90	86	85–86	82	82–82	78	78–79	75	74–75
24	83	83–84	95	95–95	91	91–91	87	87–87	83	83–84	79	79–80	76	75–76
36	79	79–80	96	95–96	91	91–92	87	87–88	83	83–84	80	79–80	76	75–77
48	76	76–76	96	95–96	91	91–92	87	87–88	83	83–84	80	79–80	76	75–76
60	73	72–73	96	95–96	91	91–92	87	87–88	83	83–84	79	78–80		
72	69	69–70	95	95–96	91	91–92	87	86–88	83	82–84				
84	66	66–67	96	95–96	91	91–92	87	86–87						
96	63	63–64	95	95–96	91	90–91								
**LOW-RISK RCC**														
12	97	97–97	97	97–97	94	94–94	91	91–91	88	88–88	85	85–85	82	81–82
24	94	94–94	97	97–97	94	94–94	91	90–91	87	87–88	84	83–84	81	80–81
36	91	91–92	97	97–97	93	93–94	90	90–90	86	86–87	83	83–84	80	79–80
48	88	88–89	97	96–97	93	93–93	89	89–90	86	85–86	82	82–83	79	78–80
60	85	85–86	96	96–97	93	92–93	89	88–89	85	85–86	82	81–82		
72	82	82–83	96	96–96	92	92–93	89	88–89	85	84–85				
84	79	79–79	96	96–96	92	92–93	88	87–89						
96	76	75–76	96	96–96	92	91–93								
**HIGH-RISK RCC**														
12	91	90–91	92	91–92	85	84–85	79	78–80	73	72–74	68	67–69	63	62–65
24	83	82–84	92	92–93	86	85–87	80	79–81	74	73–75	69	68–71	65	63–66
36	77	76–77	93	93–94	86	85–87	80	79–81	75	74–76	70	69–72	65	63–66
48	71	71–72	92	92–93	86	85–87	81	79–82	75	74–77	69	68–71	64	62–66
60	66	65–67	93	92–94	87	86–88	82	80–83	75	73–77	70	67–72		
72	61	60–63	94	93–94	88	86–89	81	79–82	75	72–77				
84	57	56–59	94	92–95	86	84–88	80	77–82						
96	54	53–55	92	91–94	85	83–88								
**mRCC**														
12	43	42–44	63	62–65	45	44–47	34	33–36	27	25–29	22	21–24	19	17–20
24	27	26–28	72	70–74	54	52–56	43	41–45	35	33–37	30	27–32	25	23–28
36	19	19–20	75	73–78	60	57–62	49	46–52	41	38–44	35	32–39	31	28–34
48	15	14–15	79	76–82	65	61–68	55	51–58	47	43–51	41	37–45	37	33–42
60	12	11–12	82	79–85	69	65–73	59	55–64	52	47–57	47	42–52		
72	9	9–10	84	81–88	73	68–77	64	58–69	58	51–64				
84	8	7–9	86	82–90	76	70–81	68	61–76						
96	7	6–8	88	83–93	80	72–87								

Subgroup analysis suggested that similar tendencies could be found in low-risk and high-risk RCC patients (Figures 2B,C). Although the COS of low-risk patients was relatively higher than that of high-risk patients, the differences were small at the beginning. Then, the differences increase gradually over time. For example, the 2 year COS in low-risk patients ranged from 92 to 94%, which had a small difference compared with that of high-risk patients (85–87%). The differences were moderate when it came to the 4 year COS between the two groups. In low-risk or high-risk patients, the COS of surviving to 5 years after living 1, 2, 3, and 4 years were 88, 91, 93, and 97% and 73, 80, 86, and 92%, respectively (higher than the observed 5 year OS: 85 and 66%).

However, things have changed quite a bit in patients with metastatic disease (Figure 2D). First, the COS was much worse than that of other patients. Second, the COS improved continuously with survivorship increasing in these patients (Figure 3B). For instance, the 2 year COS after living 1, 2, 3, 4, 5, and 6 years were 45, 54, 60, 65, 69, and 73, respectively (much higher than the observed 2 year OS: 27%). Third, the COS of these patients was much higher than the relative OS. Compared with OS, the better improvement of COS could be observed with survivorship increasing. The COS of surviving to 5 years after living 1, 2, 3, and 4 years were 27, 43, 60, and 79, respectively (much higher than the observed 5 year OS: 12%). Similar results could also be found in ccRCC patients (Table S1; Figure S2).

### CS in the FUSCC Cohort

Despite the size of FUSCC cohort being much smaller than that of the SEER cohort, similar trends could also be found (Figure 3C; Figure S3). As shown in Table S2, the overall COS in the FUSCC cohort was a litter higher than that of the SEER cohort due to more patients in the low-risk group. In addition, the differences of COS between the low-risk and high-risk patients in the FUSCC cohort were more obvious than that of the SEER cohort (Figure 3D). In metastatic patients, the COS of surviving to 5 years after living 1, 2, 3, and 4 years were 20, 32, 54, and 71%, respectively (higher than the observed 5 year OS: 13%). The improvement of COS increased over time, which was similar in the SEER cohort. The 2 year COS after living 1, 2, 3, and 4 years were 37, 46, 54, and 71%, respectively.

## Discussion

Despite RCC being not sensitive to radiotherapy and chemotherapy, most patients with RCC can live more than 5 years after diagnosis owing to the indolent nature of RCC (11, 12). Hence, traditional OS is not applicable to these patients who have survived a period of time. CS analysis is a clinically powerful measure that provides more relevant prognostic information for patients after surviving some additional years. Its usefulness was highlighted in many other malignancies, including colon, breast, and lung cancers (13–16). Few studies have investigated the relevance of CS and hazards of various causes of death in RCC patients. As both of them were useful in patient surveillance planning and their prognosis consulting, we investigate the CS and CSS of RCC patients.

Our results suggested that hazards of other causes-related death were always higher than that of cancer-specific death in low-risk RCC patients, but lower in mRCC patients. The results remain stable in different pathological types of RCC. However, hazards of other causes-related death exceeded that of cancer-specific death by 8 years in high-risk RCC in general. In addition, the time was longer in ccRCC patients. From the above results, specific follow-up strategies according to different risk and pathological types were needed. Patients with high-risk disease required longer surveillance duration (3 years longer than the expected 5 years). In addition, these patients (high-risk) with ccRCC may need longer follow-up than patients with chromophobe RCC and papillary RCC.

Another point to note from the present results was the changes of COS according to different risk patients. Previous studies found that the prognosis of RCC patients improved with additional year survived. Our study gave more precise results due to our subgroup analysis. Overall, the probability of surviving another 5 years were almost the same, after surviving 1, 2, 3, 4, and 5 years (78, 79, 80, 80, and 79%, respectively), which did not improve significantly with survivorship increasing (increased by +5, +6, +7, +7, and 6%, respectively, compared to the observed 5 year OS). Patients with low-risk and high-risk RCC had similar results. Additionally, the COS of high-risk RCC was markedly higher than that of low-risk RCC after living 4 years. Only in mRCC patients, the COS improved with survivorship increasing. After surviving 1, 2, 3, 4, and 5 years, the 5-year COS increased by +10, +18, +23, +29, and 35% (the observed 5 year OS: 12%), respectively. We can easily find that the improvement of COS was more marked in mRCC patients. The trends could also be confirmed in ccRCC patients. Therefore, these useful COS may be more suitable for patients with mRCC rather than other RCC patients.

In addition, external validation set from the FUSCC database also confirmed these findings from the SEER database. Two independent sets made our results more reliable. Based on our findings, the main clinical message is that the COS of patients with mRCC improved with survivorship increasing. CS can better help patients with mRCC and their families emotionally and psychologically because it can generate optimism for these patients with expected poorer prognosis at diagnosis. These findings also suggested that continued surveillance after 5 years may be of potential value in patients with high risk, especially in ccRCC.

It must be admitted that follow-up should be individualized based on patient requirements. There is no single surveillance program that is appropriate for all patients (3, 17, 18). The present study quantified yearly risks of death due to RCC vs. other causes and then calculated COS after diagnosis. The results help to identify subsets of RCC patients who require longer follow-up.

This study has a few limitations. First, the study was restricted by its retrospective nature like all observational studies. Similarly, the SEER database may also have the possibility of coding errors or erroneous data. However, such limitations are applicable to all previously reported studies using the SEER database (19, 20). In addition, the sample size of the FUSCC cohort was smaller than that of the SEER cohort. Some subgroup analysis, such as different pathological types of RCC, could not be fully verified.

In conclusion, the present study investigated the risks of death due to RCC vs. other causes and then calculated COS after diagnosis. The results indicate that patients with high-risk/mRCC, especially ccRCC, may need longer follow-up. Additionally, COS can better help patients with mRCC rather than other RCC patients. COS brings optimism for mRCC patients with expected poorer prognosis psychologically. Further studies are also required to verify our results and refine follow-up strategies.

## Data Availability

Publicly available datasets were analyzed in this study. This data can be found here: https://seer.cancer.gov/.

## Author Contributions

DY and YZ contributed to the conception and design. FW, MA, and JW contributed to the collection and assembly of data. NS and FW contributed to the data analysis and interpretation. NS wrote the manuscript. All authors gave their final approval of the manuscript.

### Conflict of Interest Statement

The authors declare that the research was conducted in the absence of any commercial or financial relationships that could be construed as a potential conflict of interest.

## References

[1] Molina AM Motzer RJ. Clinical practice guidelines for the treatment of metastatic renal cell carcinoma: today and tomorrow. Oncologist. (2011) 16(Suppl. 2):45–50. 10.1634/theoncologist.2011-S2-45 21346039 PMC3867940

[2] Meskawi M Sun M Trinh QD Bianchi M Hansen J Tian Z. A review of integrated staging systems for renal cell carcinoma. Eur Urol. (2012) 62:303–14. 10.1016/j.eururo.2012.04.049 22575911

[3] Escudier B Porta C Schmidinger M Algaba F Patard JJ Khoo V. Renal cell carcinoma: ESMO Clinical Practice Guidelines for diagnosis, treatment and follow-up. Ann Oncol. (2014) 25(Suppl. 3):49–56. 10.1093/annonc/mdu259 22997456

[4] Chang X Zhang F Liu T Yang R Ji C Zhao X. Comparative efficacy and safety of first-line treatments in patients with metastatic renal cell cancer: a network meta-analysis based on phase 3 RCTs. Oncotarget. (2016) 7:15801–10. 10.18632/oncotarget.7511 26908455 PMC4941278

[5] Motzer RJ Jonasch E Agarwal N Bhayani S Bro WP Chang SS. Kidney Cancer, Version 2.2017, NCCN Clinical practice guidelines in oncology. J Natl Compr Canc Netw. (2017) 15:804–34. 10.6004/jnccn.2017.0100 28596261

[6] Abdollah F Sun M Suardi N Gallina A Bianchi M Tutolo M. Prediction of functional outcomes after nerve-sparing radical prostatectomy: results of conditional survival analyses. Eur Urol. (2012) 62:42–52. 10.1016/j.eururo.2012.02.057 22421080

[7] Shigeta K Kikuchi E Hagiwara M Ando T Mizuno R Abe T. The conditional survival with time of intravesical recurrence of upper tract urothelial carcinoma. J Urol. (2017) 198:1278–85. 10.1016/j.juro.2017.06.073 28634017

[8] Kang M Park JY Jeong CW Hwang EC Song C Hong SH. Changeable conditional survival rates and associated prognosticators in patients with metastatic renal cell carcinoma receiving first line targeted therapy. J Urol. (2018) 200:989–95. 10.1016/j.juro.2018.06.030 29940249

[9] Cucchetti A Piscaglia F Cescon M Ercolani G Terzi E Bolondi L. Conditional survival after hepatic resection for hepatocellular carcinoma in cirrhotic patients. Clin Cancer Res. (2012) 18:4397–405. 10.1158/1078-0432.CCR-11-2663 22745107

[10] Kim Y Margonis GA Prescott JD Tran TB Postlewait LM Maithel SK. Curative surgical resection of adrenocortical carcinoma: determining long-term outcome based on conditional disease-free probability. Ann Surg. (2017) 265:197–204. 10.1097/SLA.0000000000001527 28009746 PMC4974140

[11] Takyar S Diaz J Sehgal M Sapunar FPandha H. First-line therapy for treatment-naive patients with advanced/metastatic renal cell carcinoma: a systematic review of published randomized controlled trials. Anticancer Drugs. (2016) 27:383–97. 10.1097/CAD.0000000000000335 26886011

[12] Edwards SJ Wakefield V Cain P Karner C Kew K Bacelar M. Axitinib, cabozantinib, everolimus, nivolumab, sunitinib and best supportive care in previously treated renal cell carcinoma: a systematic review and economic evaluation. Health Technol Assess. (2018) 22:1–278. 10.3310/hta22060 29393024 PMC5817410

[13] van Erning FN van Steenbergen LN Lemmens VE Rutten HJ Martijn H van Spronsen DJ. Conditional survival for long-term colorectal cancer survivors in the Netherlands: who do best? Eur J Cancer. (2014) 50:1731–9. 10.1016/j.ejca.2014.04.009 24814358

[14] Kim W Lee HY Jung SH Woo MA Kim HK Choi YS. Dynamic prognostication using conditional survival analysis for patients with operable lung adenocarcinoma. Oncotarget. (2017) 8:32201–11. 10.18632/oncotarget.12920 27793026 PMC5458278

[15] Paik HJ Lee SK Ryu JM Park S Kim I Bae SY. Conditional disease-free survival among patients with breast cancer. Medicine. (2017) 96:e5746. 10.1097/MD.0000000000005746 28072715 PMC5228675

[16] van Maaren MC Strobbe LJA Smidt ML Moossdorff M Poortmans PMPSiesling S. Ten-year conditional recurrence risks and overall and relative survival for breast cancer patients in the Netherlands: taking account of event-free years. Eur J Cancer. (2018) 102:82–94. 10.1016/j.ejca.2018.07.124 30144661

[17] Novara G Ficarra V Antonelli A Artibani W Bertini R Carini M. Validation of the 2009 TNM version in a large multi-institutional cohort of patients treated for renal cell carcinoma: are further improvements needed? Eur Urol. (2010) 58:588–95. 10.1016/j.eururo.2010.07.006 20674150

[18] Rini BI Stenzl A Zdrojowy R Kogan M Shkolnik M Oudard S. IMA901, a multipeptide cancer vaccine, plus sunitinib versus sunitinib alone, as first-line therapy for advanced or metastatic renal cell carcinoma (IMPRINT): a multicentre, open-label, randomised, controlled, phase 3 trial. Lancet Oncol. (2016) 17:1599–611. 10.1016/S1470-2045(16)30408-9 27720136

[19] Kim EThomas CR Jr Conditional survival of malignant thymoma using national population-based surveillance, epidemiology, and end results (SEER) registry (1973–2011). J Thorac Oncol. (2015) 10:701–7. 10.1097/JTO.0000000000000472 25590603

[20] Adam MA Thomas S Roman SA Hyslop TSosa JA. Rethinking the current American Joint Committee on Cancer TNM staging system for medullary thyroid cancer. JAMA Surg. (2017) 152:869–76. 10.1001/jamasurg.2017.1665 28636692 PMC5710458

