# Supplementary material for: Causes of Death and Conditional Survival of Renal Cell Carcinoma
Source: Front Oncol. 2019 Jul 15;9:591. doi: 10.3389/fonc.2019.00591 (PMC6644417; doi:10.3389/fonc.2019.00591)
Supplement: Supplementary file 1 [file Data_Sheet_1.docx]

**Figure Legends**

Figure S1. Hazards of death curve of RCC.

Figure S1 contains yearly hazards of death due to RCC, other causes, and any cause in low-risk RCC patients (A). Yearly hazards of death due to RCC, other causes, and any cause in high-risk RCC patients (B). Yearly hazards of death due to RCC, other causes, and any cause in metastatic RCC patients (C). Yearly hazards of death due to RCC, other causes, and any cause in different histologic subtypes in high-risk RCC (D).

Figure S2. Conditional overall survival curves of RCC.

Figure S2 contains conditional overall survival curves according to the number of years after diagnosis for all patients with ccRCC in SEER cohort (A). Conditional overall survival curves for patients with low-risk ccRCC in SEER cohort (B). Conditional overall survival curves for patients with high-risk ccRCC in SEER cohort (C). Conditional overall survival curves for patients with metastatic ccRCC in SEER cohort (D).

Figure S3. Conditional overall survival curves of RCC.

Figure S3 contains conditional overall survival curves according to the number of years after diagnosis for all patients with RCC in FUSCC cohort (A). Conditional overall survival curves for patients with low-risk RCC in FUSCC cohort (B). Conditional overall survival curves for patients with high-risk RCC in FUSCC cohort (C). Conditional overall survival curves for patients with metastatic RCC in FUSCC cohort (D).
