# Supplementary material for: Causes of Death and Conditional Survival of Renal Cell Carcinoma
Source: Front Oncol. 2019 Jul 15;9:591. doi: 10.3389/fonc.2019.00591 (PMC6644417; doi:10.3389/fonc.2019.00591)
Supplement: Supplementary file 2 [file Data_Sheet_2.docx]

Table S1 Conditional Overall Survival of ccRCC at Various Time Points

|  | | |  | |  | COS since Time Point (months) | | | | | | | | | | | | | | | | | | | | |  |  |
| --- | --- | --- | --- | --- | --- | --- | --- | --- | --- | --- | --- | --- | --- | --- | --- | --- | --- | --- | --- | --- | --- | --- | --- | --- | --- | --- | --- | --- |
|  | | | | Observed Survival | |  | 12 | |  | | 24 | |  | 36 | |  | | 48 | |  | | 60 | |  | | 72 | | |
| Time Point | | % | | 95% CI |  | % | 95% CI | | % | | 95% CI | % | | 95% CI | | % | | 95% CI | | % | | 95% CI | | % | | 95% CI | |  |
| 12 |  | 92 | | 92-93 |  | 95 | 94-95 | | 90 | | 90-91 | 86 | | 86-87 | | 83 | | 82-83 | | 79 | | 79-80 | | 76 | | 75-76 | |  |
| 24 |  | 87 | | 87-88 |  | 95 | 95-96 | | 91 | | 91-92 | 87 | | 87-88 | | 84 | | 83-84 | | 80 | | 79-80 | | 76 | | 76-77 | |  |
| 36 |  | 83 | | 83-84 |  | 96 | 96-96 | | 92 | | 91-92 | 88 | | 87-88 | | 84 | | 83-84 | | 80 | | 79-81 | | 76 | | 75-77 | |  |
| 48 |  | 80 | | 79-80 |  | 96 | 95-96 | | 92 | | 91-92 | 87 | | 87-88 | | 84 | | 83-84 | | 80 | | 79-80 | | 76 | | 74-77 | |  |
| 60 |  | 76 | | 76-77 |  | 96 | 95-96 | | 91 | | 91-92 | 87 | | 87-88 | | 83 | | 82-84 | | 79 | | 78-80 | |  | |  | |  |
| 72 |  | 73 | | 73-74 |  | 95 | 95-96 | | 91 | | 91-92 | 87 | | 86-88 | | 82 | | 81-83 | |  | |  | |  | |  | |  |
| 84 |  | 70 | | 69-70 |  | 96 | 95-96 | | 91 | | 90-92 | 86 | | 85-87 | |  | |  | |  | |  | |  | |  | |  |
| 96 |  | 67 | | 66-67 |  | 95 | 95-96 | | 90 | | 89-91 |  | |  | |  | |  | |  | |  | |  | |  | |  |
| Low-risk ccRCC | |  | |  |  |  |  | |  | |  |  | |  | |  | |  | |  | |  | |  | |  | |  |
| 12 |  | 98 | | 98-98 |  | 98 | 98-98 | | 95 | | 95-95 | 92 | | 92-93 | | 89 | | 89-90 | | 86 | | 86-87 | | 83 | | 82-83 | |  |
| 24 |  | 96 | | 95-96 |  | 97 | 97-97 | | 94 | | 94-95 | 91 | | 91-92 | | 88 | | 88-89 | | 85 | | 84-85 | | 82 | | 81-82 | |  |
| 36 |  | 93 | | 93-93 |  | 97 | 97-97 | | 94 | | 94-94 | 91 | | 91-91 | | 87 | | 87-88 | | 84 | | 83-85 | | 81 | | 80-81 | |  |
| 48 |  | 90 | | 90-91 |  | 97 | 97-97 | | 94 | | 93-94 | 90 | | 89-90 | | 86 | | 86-87 | | 83 | | 82-84 | | 79 | | 78-80 | |  |
| 60 |  | 87 | | 87-88 |  | 97 | 96-97 | | 93 | | 92-93 | 89 | | 89-90 | | 86 | | 85-86 | | 82 | | 81-83 | |  | |  | |  |
| 72 |  | 84 | | 84-85 |  | 96 | 96-96 | | 92 | | 92-93 | 89 | | 88-89 | | 85 | | 84-86 | |  | |  | |  | |  | |  |
| 84 |  | 81 | | 81-82 |  | 96 | 96-97 | | 92 | | 92-93 | 88 | | 87-89 | |  | |  | |  | |  | |  | |  | |  |
| 96 |  | 78 | | 77-79 |  | 96 | 95-97 | | 92 | | 91-93 |  | |  | |  | |  | |  | |  | |  | |  | |  |
| High-risk ccRCC | |  | |  |  |  |  | |  | |  |  | |  | |  | |  | |  | |  | |  | |  | |  |
| 12 |  | 93 | | 92-93 |  | 92 | 91-93 | | 85 | | 84-86 | 79 | | 78-80 | | 74 | | 72-75 | | 69 | | 67-70 | | 64 | | 62-65 | |  |
| 24 |  | 85 | | 84-86 |  | 92 | 92-93 | | 86 | | 85-87 | 80 | | 79-81 | | 75 | | 73-76 | | 69 | | 68-71 | | 65 | | 63-67 | |  |
| 36 |  | 79 | | 78-80 |  | 93 | 93-94 | | 87 | | 85-86 | 81 | | 79-82 | | 75 | | 74-77 | | 70 | | 68-72 | | 65 | | 63-67 | |  |
| 48 |  | 74 | | 73-75 |  | 93 | 92-94 | | 86 | | 85-88 | 81 | | 79-82 | | 75 | | 73-77 | | 69 | | 67-72 | | 63 | | 61-66 | |  |
| 60 |  | 68 | | 67-69 |  | 93 | 92-94 | | 87 | | 85-88 | 81 | | 79-83 | | 75 | | 72-77 | | 69 | | 66-72 | |  | |  | |  |
| 72 |  | 64 | | 62-65 |  | 93 | 92-95 | | 87 | | 85-89 | 80 | | 78-83 | | 74 | | 70-77 | |  | |  | |  | |  | |  |
| 84 |  | 59 | | 58-61 |  | 93 | 92-95 | | 86 | | 84-88 | 79 | | 76-82 | |  | |  | |  | |  | |  | |  | |  |
| 96 |  | 55 | | 54-57 |  | 92 | 90-94 | | 84 | | 81-87 |  | |  | |  | |  | |  | |  | |  | |  | |  |
| Metastatic ccRCC | |  | |  |  |  |  | |  | |  |  | |  | |  | |  | |  | |  | |  | |  | |  |
| 12 |  | 56 | | 54-57 |  | 67 | 65-69 | | 50 | | 48-52 | 38 | | 36-40 | | 30 | | 28-32 | | 25 | | 23-27 | | 21 | | 19-23 | |  |
| 24 |  | 37 | | 36-39 |  | 75 | 73-77 | | 57 | | 54-59 | 45 | | 42-48 | | 37 | | 34-40 | | 31 | | 28-34 | | 27 | | 24-30 | |  |
| 36 |  | 28 | | 27-29 |  | 75 | 73-78 | | 60 | | 57-64 | 49 | | 45-53 | | 41 | | 37-45 | | 36 | | 32-40 | | 32 | | 28-36 | |  |
| 48 |  | 21 | | 20-22 |  | 80 | 77-83 | | 65 | | 61-69 | 54 | | 50-59 | | 48 | | 43-53 | | 42 | | 37-48 | | 39 | | 33-44 | |  |
| 60 |  | 17 | | 16-18 |  | 81 | 77-85 | | 68 | | 63-73 | 60 | | 54-65 | | 53 | | 47-59 | | 49 | | 42-55 | |  | |  | |  |
| 72 |  | 14 | | 12-15 |  | 84 | 79-88 | | 74 | | 68-79 | 65 | | 58-72 | | 60 | | 52-68 | |  | |  | |  | |  | |  |
| 84 |  | 11 | | 10-13 |  | 88 | 82-93 | | 78 | | 70-85 | 71 | | 63-80 | |  | |  | |  | |  | |  | |  | |  |
| 96 |  | 10 | | 9-11 |  | 89 | 82-95 | | 81 | | 73-90 |  | |  | |  | |  | |  | |  | |  | |  | |  |

Table S2 Conditional Overall Survival at Various Time Points in FUSCC cohort

|  | | |  | |  | COS since Time Point (months) | | | | | | | | | | | | | | | | | | | | |  |  |
| --- | --- | --- | --- | --- | --- | --- | --- | --- | --- | --- | --- | --- | --- | --- | --- | --- | --- | --- | --- | --- | --- | --- | --- | --- | --- | --- | --- | --- |
|  | | | | Observed Survival | |  | 12 | |  | | 24 | |  | 36 | |  | | 48 | |  | | 60 | |  | | 72 | | |
| Time Point | | % | | 95% CI |  | % | 95% CI | | % | | 95% CI | % | | 95% CI | | % | | 95% CI | | % | | 95% CI | | % | | 95% CI | |  |
| 12 |  | 96 | | 95-97 |  | 96 | 95-97 | | 93 | | 92-94 | 90 | | 89-92 | | 86 | | 85-88 | | 83 | | 81-86 | | 80 | | 77-82 | |  |
| 24 |  | 93 | | 91-94 |  | 96 | 95-97 | | 94 | | 92-95 | 90 | | 88-91 | | 87 | | 85-89 | | 83 | | 80-85 | | 78 | | 75-82 | |  |
| 36 |  | 89 | | 88-91 |  | 97 | 96-98 | | 93 | | 92-95 | 90 | | 89-92 | | 86 | | 83-88 | | 81 | | 78-85 | | 74 | | 69-78 | |  |
| 48 |  | 87 | | 85-88 |  | 96 | 94-97 | | 93 | | 91-94 | 88 | | 86-91 | | 84 | | 80-87 | | 76 | | 71-80 | | 73 | | 68-78 | |  |
| 60 |  | 83 | | 81-85 |  | 97 | 95-98 | | 92 | | 90-95 | 87 | | 84-91 | | 79 | | 75-84 | | 76 | | 71-81 | |  | |  | |  |
| 72 |  | 80 | | 78-82 |  | 96 | 94-98 | | 90 | | 87-94 | 82 | | 77-86 | | 79 | | 73-84 | |  | |  | |  | |  | |  |
| 84 |  | 77 | | 74-79 |  | 95 | 92-97 | | 86 | | 81-90 | 82 | | 77-87 | |  | |  | |  | |  | |  | |  | |  |
| 96 |  | 73 | | 69-76 |  | 90 | 86-95 | | 87 | | 82-92 |  | |  | |  | |  | |  | |  | |  | |  | |  |
| Low-risk RCC | |  | |  |  |  |  | |  | |  |  | |  | |  | |  | |  | |  | |  | |  | |  |
| 12 |  | 100 | | 99-100 |  | 99 | 99-100 | | 98 | | 97-99 | 96 | | 95-97 | | 94 | | 93-95 | | 92 | | 90-93 | | 88 | | 85-90 | |  |
| 24 |  | 99 | | 99-100 |  | 99 | 98-99 | | 97 | | 96-98 | 95 | | 93-96 | | 92 | | 91-94 | | 88 | | 86-91 | | 84 | | 81-87 | |  |
| 36 |  | 98 | | 97-98 |  | 99 | 98-99 | | 96 | | 95-97 | 94 | | 92-95 | | 90 | | 87-92 | | 85 | | 82-89 | | 77 | | 72-81 | |  |
| 48 |  | 96 | | 95-97 |  | 97 | 96-99 | | 95 | | 93-97 | 91 | | 88-93 | | 87 | | 83-90 | | 78 | | 73-82 | | 75 | | 69-80 | |  |
| 60 |  | 94 | | 92-95 |  | 98 | 96-99 | | 93 | | 91-96 | 89 | | 86-92 | | 80 | | 75-84 | | 77 | | 71-82 | |  | |  | |  |
| 72 |  | 91 | | 90-93 |  | 96 | 94-98 | | 91 | | 88-94 | 82 | | 77-86 | | 79 | | 73-84 | |  | |  | |  | |  | |  |
| 84 |  | 87 | | 85-90 |  | 95 | 92-98 | | 86 | | 81-90 | 82 | | 77-87 | |  | |  | |  | |  | |  | |  | |  |
| 96 |  | 83 | | 80-87 |  | 90 | 86-94 | | 86 | | 81-92 |  | |  | |  | |  | |  | |  | |  | |  | |  |
| High-risk RCC | |  | |  |  |  |  | |  | |  |  | |  | |  | |  | |  | |  | |  | |  | |  |
| 12 |  | 94 | | 91-97 |  | 91 | 87-95 | | 82 | | 77-88 | 74 | | 67-80 | | 63 | | 55-70 | | 54 | | 46-63 | | 53 | | 44-62 | |  |
| 24 |  | 86 | | 82-90 |  | 90 | 86-94 | | 81 | | 75-87 | 69 | | 61-76 | | 60 | | 50-69 | | 58 | | 48-67 | | 51 | | 40-62 | |  |
| 36 |  | 77 | | 72-83 |  | 90 | 84-95 | | 76 | | 68-84 | 66 | | 56-76 | | 64 | | 54-75 | | 57 | | 45-69 | | 57 | | 45-69 | |  |
| 48 |  | 69 | | 63-76 |  | 85 | 77-93 | | 74 | | 64-84 | 71 | | 61-82 | | 63 | | 50-76 | | 63 | | 50-76 | | 58 | | 42-73 | |  |
| 60 |  | 59 | | 52-66 |  | 87 | 77-97 | | 84 | | 73-95 | 74 | | 60-89 | | 74 | | 60-89 | | 68 | | 51-86 | | 87 | | 77-97 | |  |
| Metastatic RCC | |  | |  |  |  |  | |  | |  |  | |  | |  | |  | |  | |  | |  | |  | |  |
| 12 |  | 63 | | 56-71 |  | 61 | 51-71 | | 37 | | 27-46 | 28 | | 19-37 | | 20 | | 10-29 | | 20 | | 10-29 | | 17 | | 8-27 | |  |
| 24 |  | 39 | | 31-46 |  | 60 | 46-73 | | 46 | | 32-59 | 32 | | 17-47 | | 32 | | 17-47 | |  | |  | |  | |  | |  |
| 36 |  | 23 | | 16-30 |  | 77 | 61-92 | | 54 | | 32-76 | 54 | | 32-76 | |  | |  | |  | |  | |  | |  | |  |
| 48 |  | 18 | | 12-24 |  | 71 | 45-97 | | 71 | | 45-97 |  | |  | |  | |  | |  | |  | |  | |  | |  |
