# Supplementary figures and images for: Causes of Death and Conditional Survival of Renal Cell Carcinoma
Source: Front Oncol. 2019 Jul 15;9:591. doi: 10.3389/fonc.2019.00591 (PMC6644417; doi:10.3389/fonc.2019.00591)

A

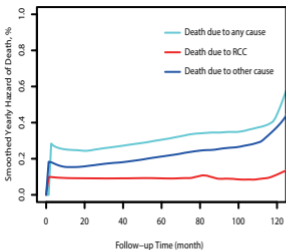

B

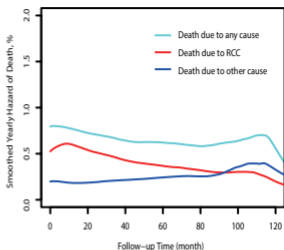

C

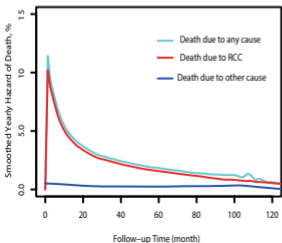

D

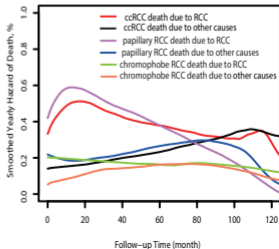

Supplement: Supplementary file 3 [file Image_1.pdf]

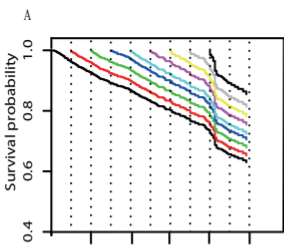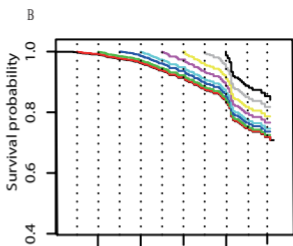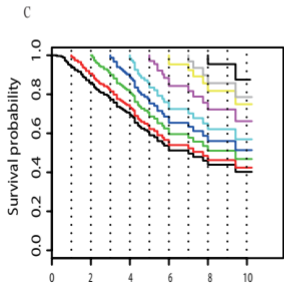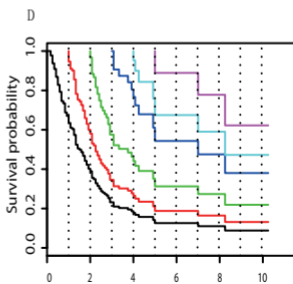

Supplement: Supplementary file 4 [file Image_2.pdf]

A

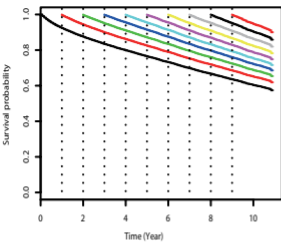

B

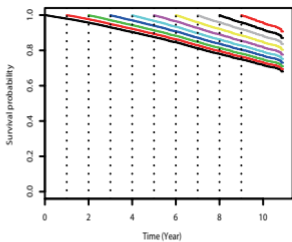

C

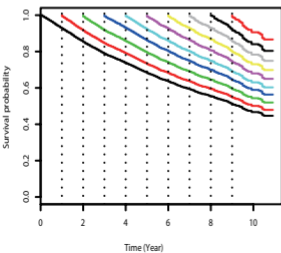

D

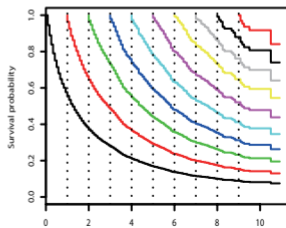

Supplement: Supplementary file 5 [file Image_3.pdf]
